# Analysis of longitudinal changes in dyspnea of patients with chronic obstructive pulmonary disease: an observational study

**DOI:** 10.1186/1465-9921-13-85

**Published:** 2012-09-25

**Authors:** Toru Oga, Mitsuhiro Tsukino, Takashi Hajiro, Akihiko Ikeda, Koichi Nishimura

**Affiliations:** 1 Department of Respiratory Care and Sleep Control Medicine, Graduate School of Medicine, Kyoto University, Kyoto, Japan; 2 Department of Respiratory Medicine, Hikone Municipal Hospital, Hikone, Japan; 3 Department of Respiratory Medicine, Tenri Hospital, Tenri, Japan; 4 Department of Respiratory Medicine, Nishi-Kobe Medical Center, Kobe, Japan; 5 Department of Respiratory Medicine, Takanohara Central Hospital, Nara, Japan

**Keywords:** COPD, Dyspnea, Airflow limitation, Diffusing capacity, Exercise, Psychological status, Disease progression

## Abstract

**Background:**

Guidelines recommend that symptoms as well as lung function should be monitored for the management of patients with chronic obstructive pulmonary disease (COPD). However, limited data are available regarding the longitudinal change in dyspnea, and it remains unknown which of relevant measurements might be used for following dyspnea.

**Methods:**

We previously consecutively recruited 137 male outpatients with moderate to very severe COPD, and followed them every 6 months for 5 years. We then reviewed and reanalyzed the data focusing on the relationships between the change in dyspnea and the changes in other clinical measurements of lung function, exercise tolerance tests and psychological status. Dyspnea with activities of daily living was assessed with the Oxygen Cost Diagram (OCD) and modified Medical Research Council dyspnea scale (mMRC), and two dimensions of disease-specific health status questionnaires of the Chronic Respiratory Disease Questionnaire (CRQ) and the St. George’s Respiratory Questionnaire (SGRQ) were also used. Dyspnea at the end of exercise tolerance tests was measured using the Borg scale.

**Results:**

The mMRC, CRQ dyspnea and SGRQ activity significantly worsened over time (p < 0.001), but the OCD did not (p = 0.097). Multiple regression analyses revealed that the changes in the OCD, mMRC, CRQ dyspnea and SGRQ activity were significantly correlated to changes in forced expiratory volume in one second (FEV_1_) (correlation of determination (r^2^) = 0.05-0.19), diffusing capacity for carbon monoxide (r^2^ = 0.04-0.08) and psychological status evaluated by Hospital Anxiety and Depression Scale (r^2^ = 0.14-0.17), although these correlations were weak. Peak Borg score decreased rather significantly, but was unrelated to changes in clinical measurements.

**Conclusion:**

Dyspnea worsened over time in patients with COPD. However, as different dyspnea measurements showed different evaluative characteristics, it is important to follow dyspnea using appropriate measurements. Progressive dyspnea was related not only to progressive airflow limitation, but also to various factors such as worsening of diffusing capacity or psychological status. Changes in peak dyspnea at the end of exercise may evaluate different aspects from other dyspnea measurements.

## Background

Dyspnea is the main symptom of which most patients with chronic obstructive pulmonary disease (COPD) complain. Guidelines recommend that symptoms as well as lung function should be monitored for the management of patients with COPD [1]. Dyspnea is regarded as a potential marker of disease progression of COPD, because it worsens over time, predicts mortality, and responds to therapy [2]. However, only a few observational studies have been performed to analyze the longitudinal changes in dyspnea [3-5]. It is still unknown how changes in dyspnea are related to changes in forced expiratory volume in one second (FEV_1_) and other clinical measurements; the gold standard measurement for following dyspnea has also not been established, as none of the available methods is optimal, having regard to their merits and limitations [6].

In previous cross-sectional studies, we reported that three dyspnea measurements with activities of daily living such as the Oxygen Cost Diagram (OCD) [7], the modified Medical Research Council dyspnea scale (mMRC) [8] and the Baseline Dyspnea Index (BDI) [9], and the two dimensions of disease-specific health status questionnaires of the Chronic Respiratory Disease Questionnaire (CRQ) [10] and the St. George’s Respiratory Questionnaire (SGRQ) [11] performed equally well in assessing dyspnea of patients with COPD; however, the Borg scale [12] at the end of exercise evaluated different aspects of dyspnea [13]. However, although unidimensional measurements such as OCD or MRC, which were initially developed to quantify dyspnea in a category or analog scale, have excellent discriminative properties, it is estimated that they would not be so useful as an evaluative instrument [14]. Therefore, we hypothesized that, although different dyspnea measurements worsened over time, the associated changes would differ depending on the instruments used, and that changes in dyspnea are related to the changes in a variety of factors, such as FEV_1_. We previously recruited patients with COPD, and assessed multiple clinical measurements every 6 months over 5 years [5,15]. In the present study, we reviewed the data and compared longitudinal changes in different dyspnea measurements and the relevant contributory factors using multiple regression analyses.

## Methods

### Subjects

We previously consecutively recruited 137 male outpatients with moderate to very severe COPD [5,15]. Inclusion criteria included: (1) smoking history of more than 20 pack years; (2) maximal FEV_1_/forced vital capacity of less than 0.7 and postbronchodilator FEV_1_ of less than 80% of the predicted normal; (3) regular attendance over 6 months; (4) no COPD exacerbations over the preceding 6 weeks; and (5) no uncontrolled comorbidities. Patient clinical measurements including smoking status, physiological measurements and patient reported measurements were evaluated at entry and thereafter every 6 months over 5 years. When a COPD exacerbation requiring a change in treatment occurred within 4 weeks of a reassessment day, the reevaluation was postponed for at least 4 weeks until the patient recovered. The study protocol was approved by the institutional ethical committee of Kyoto University.

### Physiological measurements

Pulmonary function tests were performed at least 12 hours after the withdrawal of inhaled bronchodilators [5,15]. Subjects underwent spirometry using a spirometer (AUTOSPIRO AS-600, Minato Medical Science Co. Ltd., Osaka, Japan) before and at 15 and 60 min after inhaling salbutamol (400 μg) and ipratropium bromide (80 μg). Spirometry was performed three times, and the highest values recorded were analyzed. Functional residual capacity (FRC) was measured by the closed-circuit helium method, and diffusing capacity for carbon monoxide (DL_CO_) was measured by the single-breath technique (CHESTAC-65V, Chest, Tokyo, Japan).

Exercise tests were performed 60 min after the inhalation of bronchodilators, using symptom-limited progressive cycle ergometry [5,15,16]. Exercise data were recorded using an automated exercise testing system which converts the breath-by-breath analog input into a digital form on-line. Minute ventilation (V) and oxygen tension in the expired air were determined every eight breaths, and mean V, oxygen uptake (V_2_) and carbon dioxide production were then calculated. Their peak values were the highest levels reached during exercise. Dyspnea was scored at the end of exercise using the Borg scale (0–10) [12].

### Patient reported measurements

To assess dyspnea during activities of daily living, the Japanese versions of the OCD [7,13] and the mMRC (version 1) [8,13] were used. The OCD is a visual analogue scale corresponding to oxygen requirements at different activity levels, which is represented as a value ranging from 0 to 100. mMRC is a 5-point scale (0–4) based on degrees of various physical activities that precipitate dyspnea. Less dyspnea is indicated by higher scores on the OCD, and lower scores on the mMRC.

Additionally, as a specific heath status dimension for evaluating dyspnea, the dyspnea domain of the CRQ [10,13] and the activity component of the SGRQ [11,13] were also used. Although the original CRQ version was interview-administrated, the CRQ was self-administered without informed administration in the present study. For the CRQ dyspnea, each patient defined the five items in terms of activities of daily living limited by the disease. Each item was scored on a 7-point scale, and the score was calculated as a mean of the sum. The SGRQ activity includes 16 items, and its scores range from 0 to 100. Better health is indicated by higher scores on the CRQ, and lower scores on the SGRQ.

Psychological status was evaluated using the Japanese version of the Hospital Anxiety and Depression Scale (HADS) [13,17], which consists of 14 items, 7 for anxiety and 7 for depression. Each item is scored from 0 to 3, where a score of 3 represents a state of the worst anxiety or depression. The sum of these items produces 2 subscales ranging from 0 to 21.

### Follow-up data

Among the 137 patients, 72 patients attended the last 5-year evaluation, and only one patient was unavailable for follow-up [5]. Twenty-five patients died during the 5-year period, 36 patients dropped out of the study due to an inability to attend the hospital for various reasons, and 3 patients skipped the last appointment.

### Statistical analysis

Results are expressed as means ± SE. Mixed effects models for the slopes were used to estimate longitudinal changes in the clinical parameters [5,15,18,19] using Statistical Analysis System PROC MIXED software. In the analyses, the covariates included age and smoking status as fixed effects, whereas time was entered as a random effect [5,15]. Relationships between the slope changes in dyspnea measurements and other clinical measurements were analyzed by Pearson’s correlation coefficient tests. Stepwise multiple regression analyses were used to identify those variables that could best predict the changes in dyspnea measurements. Simple linear regression analyses were performed to predict the changes in dyspnea measurements from the changes in FEV_1_. A p value of less than 0.05 was considered to indicate statistical significance, except for a p value of less than 0.01 in the case of the relationships between the slope changes.

## Results

Baseline characteristics and the annual changes in clinical measurements of the 137 male outpatients are shown in Table 1 and elsewhere [5,15]. Body mass index (BMI) significantly fell (p = 0.0013). The annual decline in postbronchodilator FEV_1_ was −25.4 ± 5.9 ml/year (p < 0.001), which was compatible with recent observational studies [20-22]. DL_CO_ significantly declined (p < 0.001), and FRC increased (p < 0.001).

**Table 1:** Baseline data and annual changes in 137 patients with COPD

	**Baseline**	**Annual change**	**P value**
Age (years)	69.0 ± 0.6		
Smoking (current/former)	34/103		
Body mass index (kg/m^2^)	21.2 ± 0.3	-0.14 ± 0.04	0.0013
Resting physiological measurements			
FEV_1_ (l)	1.22 ± 0.04	-0.0254 ± 0.0059	<0.001
FEV_1_ (%predicted)	45.9 ± 1.3	-0.9 ± 0.2	<0.001
FRC (%predicted)	110.0 ± 2.2	1.9 ± 0.4	<0.001
DL_CO_ (%predicted)	64.6 ± 1.8	-3.5 ± 0.4	<0.001
Peak exercise measurements			
Peak V_2_ (ml/min/kg)	14.8 ± 0.3	-0.5 ± 0.1	<0.001
Peak V (l/min)	40 ± 1	-2.5 ± 0.2	<0.001
Peak V (%predicted VC)	37.8 ± 0.7	-2.2 ± 0.2	<0.001
Dyspnea measurements			
OCD (0-100)	60 ± 2	-0.6 ± 0.3	0.097
mMRC (0–4)	1.1 ± 0.1	0.14 ± 0.02	<0.001
CRQ dyspnea (1–7)	5.28 ± 0.09	-0.10 ± 0.02	<0.001
SGRQ activity (0–100)	43.1 ± 1.8	1.83 ± 0.39	<0.001
Borg score at the end of exercise (0–10)	6.4 ± 0.1	-0.13 ± 0.03	<0.001
Psychological status measurements			
HADS anxiety (0–21)	4.7 ± 0.3	0.16 ± 0.08	0.046
HADS depression (0–21)	3.9 ± 0.3	0.17 ± 0.07	0.023

Data presented as means ± SE or number. *FEV*_*1*_ forced expiratory volume in one second; *FRC* functional residual capacity, *DL*_*CO*_ diffusing capacity for carbon monoxide, $\dot{V}O_{2}$ oxygen uptake, $\dot{V}E$ minute ventilation, VT tidal volume, *VC* vital capacity, *OCD* Oxygen Cost Diagram, *mMRC* modified Medical Research Council dyspnea scale, *CRQ* Chronic Respiratory Disease Questionnaire, *SGRQ* St. George’s Respiratory Questionnaire, *HADS* Hospital Anxiety and Depression Scale.

Regarding dyspnea measurements, the mMRC, CRQ dyspnea and SGRQ activity significantly worsened (p < 0.001), but the change in the OCD was not significant (p = 0.097) (Table 1). Borg score at the end of exercise rather significantly improved (p < 0.001). Regarding other clinical measurements, peak exercise measurements of V_2_, V and tidal volume (V) significantly declined (p < 0.001) and psychological status assessed by the HADS significantly worsened (p < 0.05).

Table 2 shows the inter-relationships between the changes in different dyspnea measurements. The changes in the OCD, mMRC, CRQ dyspnea and SGRQ activity were significantly moderately inter-correlated with each other (correlation coefficient (r) = 0.45-0.52, p < 0.001). The change in the Borg score was unrelated to the changes in other dyspnea measurements.

**Table 2:** Correlation coefficients between the changes in different dyspnea measurements

	**OCD (/year)**	**mMRC (/year)**	**CRQ dyspnea (/year)**	**SGRQ activity (/year)**	**Borg score (/year)**
OCD (/year)	1				
mMRC (/year)	-0.51*	1			
CRQ dyspnea (/year)	0.45*	-0.49*	1		
SGRQ activity (/year)	-0.52*	0.52*	-0.52*	1	
Borg score (/year)	-0.06	0.04	-0.10	0.02	1

* p < 0.001. *OCD* Oxygen Cost Diagram, *mMRC* modified Medical Research Council dyspnea scale, *CRQ* Chronic Respiratory Disease Questionnaire, *SGRQ* St. George’s Respiratory Questionnaire.

Table 3 shows the relationships of the changes in different dyspnea measurements to the changes in clinical measurements. Among dyspnea measurements, only the change in the CRQ dyspnea was significantly correlated with the change in BMI. The changes in the OCD, mMRC, CRQ dyspnea and SGRQ activity were significantly correlated with the changes in FEV_1_ (r = 0.29-0.49, p < 0.001) and DL_CO_ (r = 0.27-0.35, p < 0.01). These were significantly correlated with some of the changes in peak exercise measurements such as V (r = 0.22-0.30, p < 0.01). The changes in the OCD, mMRC, CRQ dyspnea and SGRQ activity were all significantly correlated to the changes in psychological status by the HADS (r = 0.33-0.46, p < 0.001). The change in the Borg score at the end of exercise was unrelated to any of the changes in clinical variables.

**Table 3:** Correlation coefficients between the changes in dyspnea and the changes in clinical variables

	**OCD (/year)**	**mMRC (/year)**	**CRQ dyspnea (/year)**	**SGRQ activity (/year)**	**Borg score (/year)**
Body mass index (kg/m^2^/year)	0.10	-0.16	0.28*	-0.19	0.14
Resting physiological measurements					
FEV_1_ (ml/year)	0.30*	-0.35*	0.36*	-0.47*	-0.08
FEV_1_ (%predicted/year)	0.29*	-0.37*	0.37*	-0.49*	-0.10
FRC (%predicted/year)	0.08	-0.03	-0.18	-0.10	0.11
DL_CO_ (%predicted/year)	0.27*	-0.35*	0.32*	-0.27*	0.03
Peak exercise measurements					
Peak V_2_ (ml/min/kg/year)	0.25*	-0.35*	0.16	-0.21	0.07
Peak V (l/min/year)	0.27*	-0.30*	0.22*	-0.24*	0.08
Peak V (%predicted VC/year)	0.14	-0.26*	0.21	-0.30*	0.05
Psychological status measurements					
HADS anxiety (/year)	-0.36*	0.36*	-0.33*	0.41*	-0.00
HADS depression (/year)	-0.45*	0.46*	-0.45*	0.43*	0.07

* p < 0.01. *FEV*_*1*_ forced expiratory volume in one second; *FRC* functional residual capacity, *DL*_*CO*_ diffusing capacity for carbon monoxide, $\dot{V}O_{2}$ oxygen uptake, $\dot{V}E$ minute ventilation, VT tidal volume, *VC* vital capacity, *HADS* Hospital Anxiety and Depression Scale, *OCD* Oxygen Cost Diagram, *mMRC* modified Medical Research Council dyspnea scale, *CRQ* Chronic Respiratory Disease Questionnaire, *SGRQ* St. George’s Respiratory Questionnaire.

Table 4 shows the results of the stepwise multiple regression analyses to best predict the changes in dyspnea measurements from the OCD, mMRC, CRQ dyspnea and SGRQ activity using the factors significantly correlated in Table 3 as explanatory variables. The changes in FEV_1_ and DL_CO_ accounted for a significant amount of the variance (correlation of determination (r^2^) = 0.05-0.19, and r^2^ = 0.04-0.08, respectively), as did the changes in the HADS anxiety or depression (r^2^ = 0.14-0.17). The change in the peak V_2_ was significantly correlated with the change in the mMRC (r^2^ = 0.07).

**Table 4:** Results of stepwise regression analyses to best predict the changes in dyspnea

	**OCD (/year)**	**mMRC (/year)**	**CRQ dyspnea (/year)**	**SGRQ activity (/year)**
Body mass index (kg/m^2^/year)			-	
FEV_1_ (ml/year)	0.05	0.06	0.08	0.19
DL_CO_ (%predicted/year)	0.05	0.08	0.07	0.04
Peak V_2_ (ml/min/kg/year)	-	0.07		
Peak V (l/min/year)	-	-	-	-
Peak V (%predicted VC/year)		-		-
HADS anxiety (/year)	-	-	-	0.14
HADS depression (/year)	0.17	0.17	0.16	-
Cumulative r^2^	0.27	0.38	0.32	0.37

Values indicate correlation of determination (r^2^). Missing values (-) indicate that independent variables were not statistically significant. *FEV*_*1*_ forced expiratory volume in one second; *FRC* functional residual capacity, *DL*_*CO*_ diffusing capacity for carbon monoxide, $\dot{V}O_{2}$ oxygen uptake, $\dot{V}E$ minute ventilation, VT tidal volume, *VC* vital capacity, *HADS* Hospital Anxiety and Depression Scale, *OCD* Oxygen Cost Diagram, *mMRC* modified Medical Research Council dyspnea scale, *CRQ* Chronic Respiratory Disease Questionnaire, *SGRQ* St. George’s Respiratory Questionnaire.

**Figure 1:**
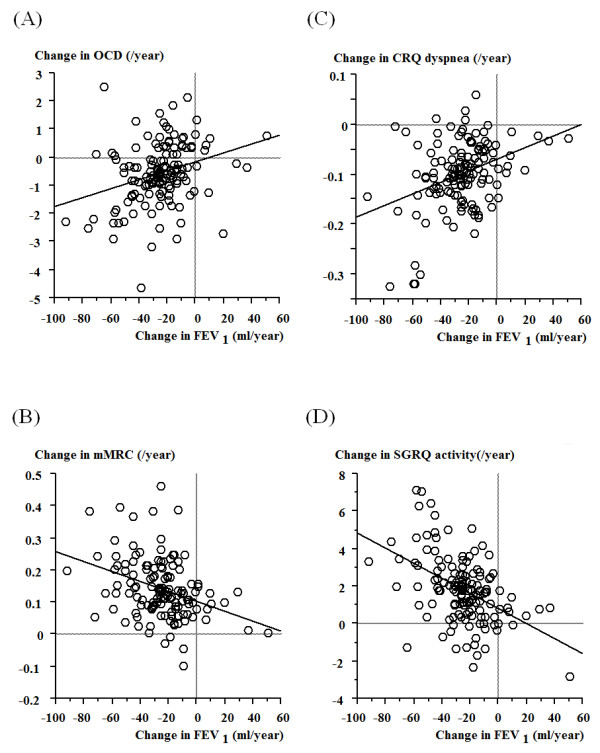
**Correlation of the annual change in FEV**_**1**_**with the annual changes in dyspnea measurements of (A) OCD, (B) mMRC, (C) CRQ dyspnea, and (D) SGRQ activity.** Positive changes in OCD and CRQ dyspnea scores indicate better dyspneic status, which are indicated by negative changes in mMRC and SGRQ activity.

Figure 1 shows the relationships between the changes in dyspnea and the change in FEV_1_. Although significant relationships were observed between the change in dyspnea and the change in FEV_1_, patients showed variable changes in dyspnea scores. Table 5 shows the results of the regression analyses. Regarding the changes in (B) mMRC, (C) CRQ dyspnea and (D) SGRQ activity, intercepts of regression lines were shifted significantly toward a worsening in dyspnea, respectively (p < 0.001).

**Table 5:** Results of linear regression analysis to predict the changes in dyspnea

	**Coefficient**	**SE**	**95% CI**	**P value**
(A) Change in OCD (/year)				
Intercept	-0.17	0.14	-0.45-0.12	0.25
Change in FEV_1_ (ml/year)	15.9	4.4	7.2-24.5	<0.001
(B) Change in mMRC (/year)				
Intercept	0.10	0.01	0.08-0.13	<0.001
Change in FEV_1_ (ml/year)	-1.55	0.35	-2.25-0.85	<0.001
(C) Change in CRQ dyspnea (/year)				
Intercept	-0.07	0.01	-0.09-0.05	<0.001
Change in FEV_1_ (ml/year)	1.17	0.26	0.66-1.68	<0.001
(D) Change in SGRQ activity (/year)				
Intercept	0.81	0.21	0.39-1.23	<0.001
Change in FEV_1_ (ml/year)	-40.3	6.5	-53.1-27.5	<0.001

*CI* confidence interval, *FEV*_*1*_ forced expiratory volume in one second, *OCD* Oxygen Cost Diagram, *mMRC* modified Medical Research Council dyspnea scale, *CRQ* Chronic Respiratory Disease Questionnaire, *SGRQ* St. George’s Respiratory Questionnaire.

## Discussion

We have shown that: 1) the mMRC, CRQ dyspnea and SGRQ activity significantly worsened over time, but not the OCD; 2) these changes were significantly correlated to the changes in various measurements such as FEV_1_, DL_CO_ and the HADS in multiple regression analyses; and 3) peak Borg score during exercise decreased over time, indicating that patients experienced significantly less breathlessness at the end of exercise, and this change was unrelated to the changes in clinical measurements.

Regarding the OCD, mMRC, CRQ dyspnea and SGRQ activity, although these exhibited similarly excellent discriminative properties [13], the present study indicated that the evaluative property of the OCD was inferior to that of other measurements. As unidimensional scales like the OCD and MRC detect the threshold of activities that produce physical limitations caused by dyspnea, they are appropriate discriminatory instruments but not satisfactory evaluative instruments [14]. Although the mMRC showed statistically significant changes, the mean change of 0.14 /year dose not reach even one grade over 5 years. The guideline recommends the MRC for dyspnea measurement in COPD for its simplicity and excellent discriminative and predictive properties [1,13,23]. However, the MRC with only 5 grades may be too broad to longitudinally detect changes in dyspnea for individuals, as other studies indicated that a multidimensional dyspnea assessment such as the Transition Dyspnea Index (TDI) [9] was superior to the MRC in terms of responsiveness [24]. Thus, some multidimensional dyspnea measurements may be preferred for longitudinal follow-ups.

When comparing between the CRQ dyspnea and SGRQ activity, considering the minimum clinically important difference (MCID) of 0.5 on the CRQ dyspnea [25] and 4 on the SGRQ [26], the SGRQ activity appeared to reach the MCID more rapidly than the CRQ dyspnea. This may be because the former incorporates more items than the latter (16 items versus 5 items). However, conversely, the more items, the more complicated and time-consuming is the measurement. Thus, an appropriate measurement should be chosen depending on the situation. As shown in Table 2, inter-relationships between the changes in the OCD, mMRC, CRQ and SGRQ were significant but modest, indicating that changes in different measurements may reflect different aspects of worsening of dyspnea.

Cross-sectionally, airflow limitation does not capture the heterogeneity of COPD including dyspnea [27]. In clinical trials assessing bronchodilator effects, improvement in FEV_1_ was significantly correlated to reduction in dyspnea [28,29]. However, no previous studies analyzed factors contributory to the changes in dyspnea longitudinally. We showed that, although cumulative correlations of determination were low and many of these factors were unknown, the change in FEV_1_ was significantly but weakly (5-19%) correlated to the changes in dyspnea. Similarly, reduction in DL_CO_ was also weakly significantly correlated to worsening of dyspnea (4-8%). Thus, as COPD is characterized by dysfunction of small airways as well as lung parenchyma [1], both may independently reflect progressive dyspnea. In contrast to the case of FEV_1_, an annual increase in FRC (one measurement of static hyperinflation) was not significantly correlated with worsening of daily dyspnea in this population. This is consistent with our cross-sectional finding that, although both airflow limitation and static hyperinflation contributed to dyspnea in patients with COPD, the former was more closely associated to dyspnea with activities of daily living than the latter [30]. The changes in peak exercise measurements were significantly correlated with the change in dyspnea in some univariate analyses, but not in multivariate analyses except for the mMRC. Considering that, in cross-sectional studies, mild correlations with airflow limitation and moderate to strong correlations with exercise capacity were expected [13], the relationships appear to be the reverse in the longitudinal studies, and further studies would be needed.

On the other hand, as compared to the physiological measurements, worsening of psychological problems such as anxiety and depression was more significantly correlated to the worsening of dyspnea. Although psychological problems are common in COPD [31] and can greatly influence dyspnea, the longitudinal inter-relationship between the two was observed in a sizable proportion (14-17%). In addition, they impact future risks of exacerbation or mortality [32,33]. Thus, it may be beneficial to target this area, with a view to reduction of progressive dyspnea in COPD.

COPD is characterized by persistent airflow limitation that is usually progressive [1]. However, recent observational studies [20-22] indicated that the rate of change in FEV_1_ is variable, and, that there are a number of patients who do not show decline of FEV_1_, who are referred to as sustainers. A wide scatter was shown in Figure 1 between the changes in dyspnea and the changes in FEV_1_, and the changes in dyspnea were considered to be as variable as in the changes in FEV_1_. Furthermore, regarding regression lines, there was a significant intercept shift in terms of deterioration of mMRC, CRQ dyspnea and SGRQ activity, indicating that these showed deterioration despite the absence of changes in FEV_1_. Similarly, Mahler et al. [3] reported that, while FEV_1_ did not increase or decrease over time, there was a small but significant deterioration in dyspnea over 2 years. Thus, disease progression occurs through a worsening in dyspnea as well as progressive airflow limitation. Clinicians should pay attention to the change in dyspnea as well as lung function, and, when it is observed, should consider possible causes.

The present multiple regression analyses have indicated that included variables were not sufficient to explain longitudinal changes in dyspnea. A limitation to this study was that we did not assess important clinical features of COPD such as exacerbations, comorbidities and systemic inflammation. Exacerbations and comorbidities can affect clinical courses of COPD including mortality and health status [34,35]. Therefore, consideration of problems critical from different angles for patients is important.

Peak Borg scores at the end of exercise were significantly reduced. This finding may be related to the observation that patients tended to stop exercise earlier due to exercise capacity deterioration as shown in peak exercise physiological indices. Alternatively, patients tended to stop exercise due rather to progressive leg fatigue than to breathlessness: however, the fact that we did not evaluate breathlessness and leg fatigue separately constitutes a limitation. In addition, changes in Borg scores were not significantly associated with any of the changes in clinical measurements, including other dyspnea measurements. In our previous studies, peak Borg scores at the end of exercise were not associated with clinical measurements cross-sectionally [13] or with mortality [36], unlike dyspnea during activities of daily living. Thus, the clinical significance of longitudinal changes in peak dyspnea during exercise remains uncertain.

We included only male patients reflecting the fact that COPD was much more common in males than females in Japan at that time. However, recent studies have reported that females showed a lower mortality rate but greater incidence of dyspnea and poor health, and more exacerbations [37,38]. Thus, there may be differences in clinical course between males and females.

## Conclusion

We have demonstrated that dyspnea during activities of daily living worsened over time in patients with COPD. As different dyspnea measurements showed different evaluative properties, we should follow dyspnea using appropriate measurements. Namely, multidimensional dyspnea measurements such as the TDI or UCSD Shortness of Breath Questionnaire [39], or dyspnea dimension of health status questionnaires such as the CRQ would be expected to be better than unidimensional measurements such as the OCD or MRC, although further studies are needed. Progressive dyspnea was related to not only progressive airflow limitation but also to various factors such as worsening of diffusing capacity or psychological status.

## Competing interests

The authors declare that they have no competing interests.

## Authors’ contributions

TO planned study design, performed statistical analyses and drafted the manuscript. KN was involved in data interpretation and critical revision of the manuscript. All authors contributed to providing care for the participants and data collection, and read and approved the final manuscript.
